# Understanding and assessing the impact of treatment in diabetes: the Treatment-Related Impact Measures for Diabetes and Devices (TRIM-Diabetes and TRIM-Diabetes Device)

**DOI:** 10.1186/1477-7525-7-83

**Published:** 2009-09-09

**Authors:** Meryl Brod, Mette Hammer, Torsten Christensen, Suzanne Lessard, Donald M Bushnell

**Affiliations:** 1 The Brod Group, 219 Julia Avenue, Mill Valley, California 94941 USA; 2 Novo Nordisk A/S, Global Development, Krogshøjvej 29, 2880 Bagsværd, Denmark; 3 Health Research Associates, 6505 216th Street SW, Suite 105, Mountlake Terrace, Washington 98043 USA

## Abstract

**Purpose:**

Diabetes is a debilitating illness requiring lifelong management. Treatments can be varied in terms of mode of administration as well as type of agent. Unfortunately, most patient reported outcome measures currently available to assess the impact of treatment are specific to diabetes type, treatment modality or delivery systems and are designed to be either a HRQoL or treatment satisfaction measure. To address these gaps, the Treatment Related Impact Measure-Diabetes and Device measures were developed. This paper presents the item development and validation of the TRIM Diabetes/Device.

**Methods:**

Patient interviews were conducted to collect the patient perspective and ensure high content validity. Interviews were hand coded and qualitatively analyzed to identify common themes. A conceptual model of the impact of diabetes medication was developed and preliminary items for the TRIM-Diabetes/Device were generated and cognitively debriefed. Validation data was collected via an on-line survey and analyzed according to an a priori statistical analysis plan to validate the overall score as well as each domain. Item level criteria were used to reduce the preliminary item pool. Next, factor analysis to identify structural domains was performed. Reliability and validity testing was then performed.

**Results:**

One hundred and five patients were interviewed in focus groups, individual interviews and for cognitive debriefing. Five hundred seven patients participated in the validation study. Factor analysis identified seven domains: Treatment Burden, Daily Life; Diabetes Management; Psychological Health; Compliance and Device Function and Bother. Internal consistency reliability coefficients of the TRIM-Diabetes/Device ranged from 0.80 and 0.94. Test-retest reliability of the TRIM-Diabetes/Device ranged from 0.71 to 0.89. All convergent and known groups validity hypotheses were met for the TRIM-Diabetes/Device total scores and sub-scales.

**Conclusion:**

Validation is an ongoing and iterative process. These findings are the first step in that process and have shown that both the TRIM-Diabetes and the TRIM-Diabetes Device have acceptable psychometric properties. Future research is needed to continue the validation process and examine responsiveness and the validity of the TRIM-Diabetes/Device in a clinical trial population.

## Introduction

Diabetes is one of the most debilitating common illnesses and requires lifelong management, often including medication, to control blood glucose levels. Treatments can be varied in terms of mode of administration (oral, syringe, pen, pump) as well as type of anti-diabetic agent (e.g., oral hypoglycemic agents, GLP-1 or insulin).

The impact of both treatment drug and treatment delivery system is multifaceted. To fully understand these impacts, the patient's perceptions of the impact of treatment on functioning and well-being must be identified and accurately assessed. Defining these impacts should cross traditional domain boundaries of health-related quality of life (HRQoL), treatment satisfaction, and treatment behavior to be truly comprehensive.

Unfortunately, most patient reported outcome (PRO) measures currently available to assess the impact of diabetes treatment are specific to Type 1 or Type 2 diabetes treatment modality or delivery systems and are designed to be either a HRQoL or a treatment satisfaction measure. As a result, they are not inclusive of all potential impacts. To address these gaps, the Treatment Related Impact Measure-Diabetes (TRIM-Diabetes), and the Treatment Related Impact Measure-Diabetes Device (TRIM-Device) measures, which capture the full range of impacts of diabetes treatment on patients' functioning and well-being across type 1 and type 2 diabetes, as well as across all currently available delivery systems and treatments (oral agents, GLP-1 pens, inhaled or pump delivered insulin and insulin delivered with syringe/pens) were developed. The development process for the TRIM-Diabetes/Device has been iterative, incorporating and synthesizing information on new delivery systems and treatments as they developed. This paper presents the item development and validation of the TRIM Diabetes/Device.

## Methods

The development of the TRIM Diabetes/Device followed draft FDA guidelines for the development of new PRO measures [1]. Ethics/IRB approval was obtained for both the item development and validation phases of the process.

### Item Development

#### Item Generation

The development of the item content for the TRIM Diabetes/Device began in 2002 with the development of the TRIAD Measures (The Diabetes Symptom Measure (DSM), Diabetes Productivity Measure (DPM) and the Diabetes Medication Satisfaction Measure (DiabMedSat)) for oral agents and injectable treatments (syringe and pen) for type 1 and 2 diabetes [2]. This knowledge was supplemented in 2006 regarding inhaled insulin and in 2008 for insulin pumps and GLP-1 pens [3]. To develop the TRIM-Diabetes, previous data from the development of the Diabetes TRIAD Measures were qualitatively re-examined and re-analyzed along with the newly collected information regarding inhaled and pump delivered insulin and thereby forming the basis for the TRIM-Diabetes/Device development project.

Information regarding the methodology for the collection of patient interview data from the TRIAD measures has been previously published [2]. Therefore only information on the data collected since 2006 are presented here. This data included: (1) telephone or in-person interviews of diabetes experts defined as endocrinologists or internists; and (2) telephone or in-person individual interviews and focus groups of type 1 and type 2 diabetes patients who had used inhaled and pump delivered insulin in either the U.S. or Australia, and is presented here. These interviews followed a semi-structured interview guide which included open-ended questions regarding the perceived impact of treatment on the social, physical, and psychological aspects of life, treatment satisfaction issues, and the specific variables that act as moderators (i.e., factors that either help or hinder the impact of treatment). Expert and individual patient telephone interviews each lasted approximately one hour. Patient focus groups lasted approximately two hours. Completed interviews were used to guide and inform subsequent interviews. Thus issues that were raised by experts and patients previously were further explored and either confirmed or rejected thereby ensuring high content validity. The number of interviews and focus groups needed to ensure content validity was determined by the 'point of saturation' (i.e., no new information appeared during the last interview/focus group). All interviews and focus groups were conducted by the first author, who is a mental health clinician and trained group facilitator. All inhaled insulin patients were recruited for the interviews by a physician who had treated them for their diabetes with inhaled insulin either currently or in the past three months. Current insulin pump patients were recruited through a professional medical marketing group from their volunteer panel. Both clinical experts and patients received an honorarium for their participation in the interviews.

Data from all interviews were coded and hand sorted and qualitatively analyzed to identify common themes and concepts. This analysis was then considered, along with the previously collected TRIAD focus group data analyses, to create a conceptual model of the multifaceted impact of diabetes medication across the spectrum of delivery systems. Based on this model, the preliminary items for the TRIM-Diabetes/Device were then generated to reflect the model domains. Domains (expected to become subscales of the final measure) were named to reflect the item content for that domain.

#### Cognitive Debriefing

Cognitive debriefing of the preliminary TRIM-Diabetes/Device measure, based on pre-defined item definitions, was conducted in an independent sample of type 1 and 2 persons with diabetes. Each method of diabetes medication as well as administration type was represented (three participants each were currently on oral medication, insulin by syringe, insulin by pen, insulin by pump or GLP-1 pen). It was not possible to include patients using inhaled insulin as it was no longer commercially available at the time of the debriefing.

Participants were mailed (or e-mailed) the TRIM-Diabetes/Device in advance and were asked to complete it prior to a prearranged individual telephone interview to assess comprehension, wording, formatting, clarity, and relevance of items. During this interview, for each item respondents were asked: 1) "What did the question mean to you?"; 2) "Was the question worded in a way that made sense to you?"; 3) "Was the question in any way offensive or objectionable to you?"; and 4) "Was the question about something which is important or relevant to you regarding your diabetes medication?" Respondents were then asked overall: 1) "Were the instructions and formatting clear?"; 2) "Did the response choices make sense?"; 3) Does a two-week recall time frame seem appropriate considering what the questions are about?; 4) "When you completed the questionnaire, did you have any difficulty accurately remembering your experiences over the past two weeks?"; 5) "Is there anything we forgot to ask?"; and 6) "Is there anything else you would like to comment on regarding the survey?"

After the first five participants were interviewed, findings were reviewed and a decision was made as to whether any changes to the measures were necessary. This process continued in blocks of five participants (one from each treatment/administration type group) until a determination was made that readability and relevance was acceptable based on consensus agreements between respondents in an entire block.

### Validation Study

#### Procedures

An online validation study was conducted to collect data to assess the measurement and psychometric properties of the TRIM-Diabetes. To be eligible for the study, the subject was required to be over the age of eighteen, currently on their diabetes treatment, and able to read and comprehend English. The sample selection process created the sampling frame of targeted persons with diabetes who went through a healthcare profiler and self-reported they had either type 1 or type 2 diabetes diagnosed by a physician. To avoid potential bias associated with panel recruitment from a single source or single methodology, a multi-sourced panel recruitment strategy was employed including permission e-mails, affiliate networks, and web site advertising. A stratified sample procedure was employed using invitation selection criteria to account for disproportional response rates between stratification categories. Stratification variables were age, ethnicity, income and primary method/type of diabetes medication (oral agents, insulin syringe, insulin pen or insulin pump, GLP-1 pen).

#### Measures

The following measures were administered in a validation survey battery:

##### The TRIM-Diabetes/Device Preliminary Version

A 60-item self-report questionnaire assessing six hypothesized domains: Productivity (Daily Activities), Productivity (Work), Psychological, Device Satisfaction, Efficacy and Burden. The five-point Likert like response options, for all items, range from Not at all/Never to Extremely/Almost always, Always or Extremely dissatisfied/inconvenient to Extremely satisfied/convenient, depending upon the item stem and are scored so that a higher score indicates a better health state.

##### Problem Areas in Diabetes (PAID)

A 20-item self-report scale developed to assess the current level of diabetes-related emotional distress both in type 1 and type 2 diabetes. PAID items contain commonly expressed negative emotions related to living with diabetes (e.g., worrying about hypoglycemia, feeling burned out by the daily efforts to manage the diabetes, feeling worried about the future and complications) that are rated on a five-point Likert scale ranging from 0 (not a problem) to 4 (a serious problem); scores are summed and standardized to a 0-100 scale, with higher scores indicating higher emotional distress [4].

##### Activity Impairment Assessment (AIA)

A five-item questionnaire assessing the amount of time that an individual's work or regular activities have been impaired as a result of their condition. Patients respond to AIA items on a five-point-type scale and are given a total score, where a higher score indicates greater impairment [5].

##### Insulin Treatment Satisfaction Questionnaire (ITSQ)

A 22-item questionnaire assessing treatment satisfaction for diabetic patients on insulin. In addition to a total score (sum of all domains), the items make up five domains: inconvenience of regimen, lifestyle flexibility, glycemic control, hypoglycemic control, and insulin delivery device satisfaction. All items are rated on a seven-point Likert scale, with the higher score (for the total score and for each subscale) indicating better treatment satisfaction. Only the inconvenience of regime domain was used in this study [6].

##### Treatment Satisfaction Questionnaire for Medication (TSQM)

A 14-item generic questionnaire that measures a patient's satisfaction with medication. Items are rated on a five- or seven-point scale according to patients' experience with the medication in terms of satisfaction, bother/interference with side effects, ease of use and confidence, with a higher score indicating greater satisfaction [7].

##### Medication Compliance Scale (MCS)

A six-item unvalidated measure assessing how often a patient thinks about postponing or skipping doses, or has actually postponed or missed doses over the past two weeks. Items are scored on a six-point Likert scale, from 0 (never) to 5 (always). The total score is calculated by summing item values with higher scores indicting greater compliance problems [8].

##### Diabetes Medication Satisfaction (DiabMedSat)

A 21-item measure consisting of three sub-scales: burden, efficacy and symptoms that was developed to measure diabetes treatment satisfaction and is applicable to a wide range of diabetes therapies. Items are rated on a five- or seven-point scale according to patients' experience with the medication, with a higher score indicating greater satisfaction [2].

##### Quality of Life Enjoyment and Satisfaction Questionnaire (Q-LES-Q) (Short Form)

A 16-item questionnaire developed to assess the degree of enjoyment and satisfaction experienced in eight areas (physical health, subjective feelings of well-being, work, household duties, school, leisure, social relationships, and general life quality). Each item is rated on a five-point Likert scale. Scores are aggregated, with higher scores indicative of greater enjoyment or satisfaction in each domain [9].

##### Center for Epidemiologic Studies Depression Scale (CES-D)

A 20-item measure comprising six scales reflecting major dimensions of depression: depressed mood, feelings of guilt and worthlessness, feelings of helplessness and hopelessness, psychomotor retardation, loss of appetite, and sleep disturbance experienced in the past week. Response categories indicate the frequency of occurrence of each item, and are scored on a four-point scale. Higher scores (both item and total scores) indicate more depressive symptoms. A score of 16 or higher has been used extensively as the cut-off point for high depressive symptoms on this scale [10].

##### Diabetes Fear of Injecting and Self-Testing Questionnaire Fear of Self Injecting subscale (D-FISQ)

A 15-item quality-of-life subscale that measures fear of self-injecting in adult diabetics. Subjects rate the items on a four-point Likert scale. Scores are summed, so that a higher score indicates greater fear [11].

### Statistical Methods

Validation procedures were conducted according to an *a priori *developed statistical analysis plan (SAP). First, item level psychometric and conceptual criteria were used to refine and reduce the preliminary item pool and reduce redundancy between items. Next, factor analysis to identify structural domains was performed. Reliability and validity testing was then performed. It is the intention of the developers that the TRIM-Diabetes/Device may be used either as a total score or that each domain can stand alone as a separate measure. Therefore, all reliability and validity tests were performed on both the total scores and for each domain.

#### Item Characteristics and Measurement Model (Scaling)

For **item reduction **both item psychometric properties and conceptual importance were taken into consideration in making retention/deletion decisions for the initial potential pool of 60 items. Items were considered for deletion, based on psychometric criteria: if the item had missing data (i.e., no response) >5% of the time; if ceiling effects were present (>50% optimal response); or if item-to-item correlations within the total item pool were high, thus indicating redundancy between items (Pearson's correlation coefficient >0.70) [12]. Items that did not perform well psychometrically could be considered for retention if conceptually important and/or unique.

The **factor structure **was determined by an exploratory principle component factor analysis using Varimax orthogonal rotation with Kaiser normalization. Although *a priori *conceptual domains were developed, the number of factors in the analysis was not specified so as not to force an inappropriate solution. A scree plot was examined to confirm the final factor solution. Item-to-total scale correlations were assessed using the Pearson's correlation between individual item scores and the total subscale score for the associated subscale. Correlation coefficients <0.40 were considered evidence of poor association [13].

#### Test for Reliability

The **internal consistency reliability **was assessed using Cronbach's alpha. This statistic is used to analyze additive scales to determine to what degree the items within the scale are associated. A high internal consistency suggests that the scale or subscale is measuring a single construct. Alpha values range from 0.00 to 1.00; however, a minimum correlation of 0.70 is preferred to claim the instrument is internally consistent [14].

The **test-retest reliability **was assessed at approximately two weeks post initial completion of the battery. To be eligible for the retest, participants had to respond "No" to the questions: "Have you experienced any major life events since you filled out the previous questionnaire approximately 2 weeks ago (e.g., moving, divorce, losing job)?" and "Has the past 2 weeks been an unusually stressful period for you?" and respond "Yes" to the question: "Have you been taking the same diabetes medication over the past 2 weeks?" An alpha of >0.70 was considered evidence of acceptable test-retest reliability.

#### Tests for Validity

The validation of the TRIM-Diabetes/Device followed the analyses as specified in the SAP. However, since the factor analyses yielded slight differences from the hypothesized domains, some of the *a priori *defined hypotheses for the validation had to be altered to fit the new measurement model. These new hypotheses were formulated after finalizing the factor structure and BEFORE examining the data for validity and reliability and have been considered as *a priori *hypotheses.

The **convergent validity **was evaluated by testing the following *a priori *hypotheses using a two-tailed Pearson's correlation coefficient with significance at the p < 0.05 level. When more than one hypothesis per domain is proposed, the minimum threshold of at least one hypothesis had to be met to claim convergent validity. Correlation coefficients >0.40 were considered acceptable evidence of moderate to strong associations [13].

H_01_: Total score: TRIM-Diabetes total will be significantly related to generic treatment satisfaction (TSQM) and/or an overall self-report total impact item.

H_02_: Treatment Burden subscale: TRIM-Diabetes Treatment Burden will be significantly related to burden (burden subscale of the DiabMedSat) and/or an overall burden self-report item.

H_03_: Daily Life subscale: TRIM-Diabetes Daily Life will be significantly related to restrictions in daily activities (AIA) and/or an overall daily life self-report item.

H_04_: Diabetes Management: TRIM-Diabetes Management will be significantly related to self-reported efficacy (Efficacy subscale of the DiabMedSat and TSQM efficacy) and/or an overall diabetes control self-report item.

H_05_: Psychological Health subscale: TRIM-Diabetes Psychological Health will be significantly related to self-reported problems with diabetes (PAID) and/or an overall emotional self-report item.

H_06_: Compliance subscale: TRIM-Diabetes Compliance will be significantly related to assessed compliance (MCS).

H_07_: Total score: TRIM-Diabetes Device total and the domains of Device Function and Device Bother subscales will be significantly related to self-reported device satisfaction (subscale of the TSQM and ITSQ) and an overall burden of medication self-report item.

The **known-groups validity**, or the ability of a PRO to distinguish between groups known to differ on characteristics which are expected to impact the PRO assessment, was evaluated by assessing the following *a priori *hypotheses. The TRIM-Diabetes scores of the known groups were compared using one-way ANOVA with groups as a fixed factor with p-values at the p < 0.05 level as evidence of a significant difference between known group. For domains with two hypotheses, at least one had to be met as the minimal threshold to claim known group validity.

H_08_: Total score: TRIM-Diabetes total will be significantly greater for those willing to switch to another medication (coded as not at all, slightly or moderately, extremely interested) or not recommend to others and/or as compliance improves.

H_09_: Treatment Burden subscale: TRIM-Diabetes Treatment Burden will significantly increase as number of daily injections increases and/or the type of treatment becomes more burdensome (would be less for orals/tablet group).

H_10_: Daily Life subscale: TRIM-Diabetes Daily Life will significantly increase as life satisfaction increases (Q-LES-Q) (coded as poor/fair/good) and/or, for those who work, greater satisfaction for those who lost fewer days from work due to diabetes (<1 day/1-2 days/3+ days).

H_11_: Diabetes Management subscale: TRIM-Diabetes Management score will significantly increase as: A1c levels improve (coded as <6.8/6.8 to 8.0/>8.0,), the number of medical visits decreases (coded as none/1/2+), change in diabetes treatment plans due to low blood sugar decreases and/or as self report diabetes control increases.

H_12_: Psychological Health subscale: TRIM-Diabetes Psychological Health will significantly increase as depression (CES-D) decreases and/or level of family and friends support of diabetes management efforts increases.

H_13_: Compliance subscale: TRIM-Diabetes Compliance will be significantly greater for those patients only taking oral medications, lower for those using either a pump, syringe, or pen.

H_14_: Device Satisfaction: TRIM-Diabetes Device total and device Function and Bother will significantly increase as fear of injections (D-FISQ) decreases (for those on any injectable treatment).

#### Interpretability: Minimally Important Difference

Since we did not have longitudinal data to examine the minimally important difference (MID) using a change score, self-report items also included in the battery, one per domain of the TRIM-Diabetes/Device, were used as anchors to approximate the MID. This analysis was considered exploratory and is meant to provide preliminary estimates of differences established using an anchor-based approach. To calculate the MID, the relationship and magnitude of change between these self-report "overall" items to the scores of each TRIM-Diabetes domain score were examined. As specified in the SAP, the MID considered changes in scores of TRIM-Diabetes domains between responses of roughly "Slightly" and "Somewhat" as the minimally important interval. For example, the difference in the mean response for the TRIM-Diabetes Burden domain score for those who respond "Slightly burdensome" and those that respond "Somewhat burdensome" on the independent item "Overall, how burdensome do you think that your insulin/diabetes medication has been?" was calculated. One-half standard deviations were calculated as the threshold for the difference to assess the MID [15].

## Results

### Item Development

Fifty-eight patients in six focus groups and nine telephone interviews were required to reach the saturation point whereby no new information was gathered regarding the treatment impact of inhaled and pump delivered insulin. This data was then combined with the information gained from the TRIAD measure interviews and a preliminary conceptual model of the impact of insulin treatment was derived directly from this analysis and synthesis. Content validity analysis of the interview transcripts found that areas of impacts were similar for both type 1 and type 2 respondents and therefore the measure could be considered appropriate for both. Based on this model the initial TRIM Diabetes/Device items were generated and underwent cognitive debriefing.

Fifteen subjects on injection, pen or pump delivered insulin, GLP-1 or oral treatments in the U.S (nine women and six men; five type 1 diabetics and ten type 2) were cognitively debriefed. Three iterations (three blocks of five participants) were required to refine the items in terms of readability and relevance and reach consensus of an entire block. As a result of the cognitive debriefing, a 60-item validation ready TRIM-Diabetes/Device was generated.

Combined, the sample for all focus groups, individual telephone interviews and cognitive debriefings included 105 participants: 28 persons with diabetes in the U.S. and U.K. were interviewed in focus groups, individual telephone interviews or cognitively debriefed for the TRIAD measures[2], and 73 persons with diabetes were interviewed in focus groups, individual telephone interviews and cognitive debriefings in the U.S. and Australia for inhaled and pump delivered insulin. Table 1 provides the patient interview sample description for all patient interviews, focus groups and cognitive debriefings used for item generation for the TRIM-Diabetes/Device.

**Table 1:** Patient Interview Sample Description

**Demographics Characteristics**	**Total**
GENDER, N (%); N = 105	
Male/Female	49 (47%)/56 (53%)

DIABETES TYPE; N = 100	
Type 1/Type 2	51 (51%)/49 (49%)

HOW LONG AGO DIAGNOSED WITH DIABETES, N (%); N = 104	
< 1 year	1 (1%)
1 - 5 years	27 (26%)
6 - 10 years	22 (21%)
> 10 years	54 (52%)

TYPE OF DIABETES TREATMENT, N (%); N = 103	
Oral/tablet	20 (19%)
Injectable insulin	15 (15%)
Pump insulin	24 (23%)
Inhaled insulin	38 (37%)
Pen insulin	3 (3%)
GLP-1	3 (3%)

AGE (Years); N = 95	
Mean (range)	49 (20-74)

EDUCATION, N (%); N = 101	
Less than or Completed High School or GED	36 (36%)
College Degree (Associate's Degree or B.A.)	40 (40%)
Graduate Degree (or higher)	25 (25%)

ETHNICITY, N (%); N = 101	
White/Caucasian	72 (71%)
Black/African American	14 (14%)
Latino/Hispanic/Mexican American	11 (11%)
Asian American/Native American/Alaskan Native/Pacific Islander	3 (3%)
Mixed Racial/Other Background	1 (1%)

MARITAL STATUS, N (%); N = 105	
Single	25 (24%)
Married/Partnered	65 (62%)
Divorced/Widowed	15 (14%)

HOUSEHOLD INCOME, N (%); N = 89	
Less than $20,000	8 (9%)
$20,000 TO $39,999	16 (18%)
$40,000 AND OVER	65 (73%)

### Validation Study

#### Sample

The final sample for validating the TRIM-Diabetes was comprised of 507 subjects. The age of the study sample ranged from 18 to 80 years, with a mean age of 51 years. The population was 53% female, 84% white, 6% African American, and 81% were living with others. About three quarters (74%) have type 2 diabetes. Table 2 provides the validation sample description details.

**Table 2:** Validation Study Sample Description

**Demographics Characteristics**	**Total** **N = 507**
GENDER, N (%)	
Male/Female	240 (47%)/267 (53%)

DIABETES TYPE, N (%)	
Type 1/Type 2	134 (26%)/373 (74%)

TYPE OF DIABETES TREATMENT, N (%)	
Oral/tablet	102 (20%)
Injectable insulin	100 (20%)
Pump insulin	101 (20%)
Inhaled insulin	102 (20%)
GLP-1	102 (20%)

HOW LONG AGO DIAGNOSED WITH DIABETES, N (%)	
< 1 year	17 (3%)
1 - 5 years	116 (23%)
5 - 10 years	133 (26%)
> 10 years	241 (48%)

LAST HEMOGLOBIN A1C VALUE, IF KNOWN N (%)	
< 6.8	107 (33%)
6.8 - 8.0	117 (36%)
> 8.0	101 (31%)

AGE (Years):	
Mean (range)	51.4 (18-80 years)
Between age 18-30	18.9%
Between age 31-50	20.9%
Between age 51-79	51.7%
Over age 70	8.5%

EDUCATION, N (%)	
Less than or Completed High School or GED	255 (50%)
College Degree (Associate's Degree or B.A.)	174 (34%)
Graduate Degree (or higher)	78 (15%)

ETHNICITY, N (%)	
White/Caucasian	427 (84%)
Black/African American	31 (6%)
Latino/Hispanic/Mexican American	22 (4%)
Native American/Alaskan Native Asian American/Pacific Islander	14 (3.2%)
Mixed Racial/Other Background	13 (3%)

MARITAL STATUS, N (%)	
Single	73 (14%)
Married/Partnered	343 (68%)
Divorced/Widowed	91 (18%)

HOUSEHOLD INCOME, N (%)	
Less than $20,000	66 (13%)
$20,000 TO $39,999	125 (25%)
$40,000 TO $59,999	108 (21%)
$60,000 TO $99,999	139 (27%)
$100,000 AND OVER	69 (14%)

#### Item Characteristics and Measurement Model (Scaling)

The response distributions showed no missing data (note this was an online data collection study not allowing for missing data). Nine items showed a ceiling effect (higher than 50%). Several pairs of items were found to be correlated at or above 0.70, indicating possible redundancy. Several items were also revealed to be unclear in their fundamental concept, and thus the items did not fit into the conceptual framework. Based on these initial indicators, 24 items were dropped from the instrument prior to performing subsequent psychometric analysis.

After the factor analyses were completed, varimax rotation (with eight iterations) determined that there were seven distinct domains, which were labeled: **Treatment Burden**, **Daily Life **(previously hypothesized as Productivity); **Diabetes Management **(previously hypothesized as Efficacy); **Psychological Health**; and a new domain labeled **Compliance**. The device satisfaction items factored into two separate domains labeled **Device Function **and **Device Bother**. It was determined that the two device domains formed their own independent measure of device satisfaction and could be considered a separate stand-alone measure of device impact (TRIM-Diabetes Device), which can be used either independently or in concert with the TRIM-Diabetes. The scree plots confirmed five factors and two factors with eigenvalues of greater than one for the TRIM-Diabetes and TRIM-Diabetes Device, respectively.

Table 3 shows the rotated component matrix result for the TRIM-Diabetes/Device scales.

**Table 3:** Factor Structure. Rotated Component Matrix

	**Component (regression coefficients)**				
	**1**	**2**	**3**	**4**	**5**

**Treatment Burden**					

Store your medication	.811				

Prepare your medication for use	.780				

Take your medication at the right time	.774				

Carry your medication and supplies around with you	.738				

The ease and convenience of your medication	.712				

Monitor your blood sugar as often as necessary	.645				

**Daily Life**					

Social activities		.749			

Do you have to limit your daily activities?		.743			

Do you accomplish less than you would like to?		.693			

Meal time planning		.675			

Do you feel tension in your relationships with friends or family?		.480			

**Diabetes Management**					

Help you prevent feeling tired or a lack of energy			.813		

Help you avoid high blood sugar (hyperglycemia)			.758		

Help you manage your weight			.754		

Help you control your diabetes			.750		

Help you avoid low blood sugar (hypoglycemia)			.683		

**Compliance**					

Miss a dose				.863	

Delay or postpone taking your medication				.825	

Take your medication at a different time than prescribed				.798	

Worry that you forgot to take/or missed your last dose of medication				.682	

**Psychological Health**					

Angry					.796

Nervous or anxious					.783

Worried about side effects from my medication					.734

Depressed					.731

Unhealthy					.721

Worried about my blood sugar control					.717

Worried that the medication is not helping to slow down or prevent complications from my diabetes					.702

Feel embarrassed or awkward when taking your medication					.401

	**Component (regression coefficients)**				

	**1**		**2**		

**Device Function**					

That you are using the device properly	.819				

Keep your device functioning properly	.797				

Ease - learn how to use your device	.764				

That your device delivers the correct, full dose of your medication	.734				

Adjust your medication for small dose changes	.677				

**Device Bother**					

Physical discomfort related to using your device			.886		

Using your device in public			.845		

Bothered-Size of your device			.818		

#### Reliability Results

Internal consistency reliability coefficients of the TRIM-Diabetes and TRIM-Diabetes Device (total score and all subscales) are all in the acceptable range from 0.80 and 0.94.

Test-retest reliability was analyzed in a subset of 56 subjects who met the time gap eligibility of two weeks plus and minus a day (13-15 days). Test-retest coefficients of the TRIM-Diabetes/Device (total score and all subscales) are in the acceptable ranging from 0.71 to 0.89.

Table 4 provides the internal consistency and test-retest reliability results.

**Table 4:** Internal Consistency and Test-Retest Reliability. Intra-class Correlation Coefficient (ICC) Statistics on the TRIM-Diabetes/Device

**Domain Subscale Identification**	**Alpha Coefficients**	**Test-Retest Reliability (n = 56)**
TRIM-Diabetes Total (28 items)	0. 94	0.85

Burden (6 items)	0.88	0.77

Daily Life (5 items)	0.86	0.75

Diabetes Management (5 items)	0.88	0.80

Compliance (4 items)	0.88	0.71

Psychological (8 items)	0.91	0.83

TRIM-Diabetes Device Total (8 items)	0.80	0.89

Device Function (5 items)	0.82	0.82

Device Bother (3 items)	0.83	0.78

*Reliability based on internal consistency using Cronbach's alpha.

#### Validity Results

All convergent validity hypotheses were met for the TRIM-Diabetes and TRIM-Device total scores and subscales.

The total TRIM-Diabetes was significantly correlated (r = 0.63) with the Global Satisfaction scale of the TSQM. The Treatment Burden domain (TRIM-Diabetes) had a significant association with the DMS Burden subscale (r = 0.45). The Daily Life subscale correlated significantly with the AIA total score (r = -0.67), while the Diabetes Management subscale had a significant correlation of 0.66 and 0.60 with the DiabMedSat Efficacy and TSQM Effectiveness scales, respectively. Finally, predictions were met with significant correlations between the TRIM-Diabetes Psychological Health and the PAID (r = -0.75) and the TRIM-Diabetes compliance and MCS (r = -0.69). As expected, significant correlations were found between the self-report item addressing impacts on life ("Overall, how much of an impact has your insulin/diabetes medication had on your life?") and the TRIM-Diabetes Total score (0.55); burden ("Overall, how burdensome do you think that your insulin/diabetes medication has been?") and the Treatment Burden domain (0.50); daily life ("Overall, how much do you think that your insulin/diabetes medication has interfered with your daily life and productivity?") and the Daily Life domain (0.57); efficacy ("Overall, how well does your insulin/diabetes medication control your diabetes?") and the Diabetes Management domain (0.61); and psychological impacts ("Overall, how does your insulin/diabetes medication impact how you feel emotionally?") and the Psychological Health domain (0.53).

The Device Function domain correlated significantly with the Convenience scale of the TSQM (r = 0.60) and the ITSQ Device Satisfaction scale (r = 0.46). The TRIM-Diabetes Device Bother domain had a significant association with the DMS Burden subscale (r = 0.63). Also as expected, significant correlations were found between the self-report item addressing medication device ("Overall, how satisfied are you with your insulin/diabetes medication device?") and the TRIM-Diabetes Device Total score (r = 0.55); and device bother ("Overall, how burdensome do you think that your insulin/diabetes medication has been?") correlated to the Device Bother score (r = 0.54).

All **known-groups validity **hypotheses were met for the TRIM-Diabetes and TRIM-Diabetes Device total scores and subscales.

The total TRIM-Diabetes was able to distinguish between willingness of respondents to change their diabetes treatment (F = 83.7, p < 0.001). There was also a significant difference between those compliant versus those not compliant with their treatment (F = 136.6, p < 0.001). While there was a positive trend, the TRIM-Diabetes Burden domain was not significant in distinguishing between the number of daily injections patients indicated; however, it was able to discriminate between the types of treatment (oral, pump and syringe, F = 27.7, p < 0.001). The Daily Life domain was able to discriminate between levels of satisfaction as measured by the Q-LES-Q (F = 47.5, p < 0.001) and days lost from work due to diabetes (F = 43.1, p < 0.001). The TRIM-Diabetes Management domain significantly distinguished between HbA_1c _levels (F = 16.6, p < 0.001), the number of medical visits (F = 4.8, p < 0.01), the changing of diabetes treatment plans (none/1-2 times/>3, F = 8.5, p < 0.001), and diabetes control (F = 115.8, p < 0.001). The Psychological Health subscale was able to discriminate between depression severity (F = 152.9, p < 0.001) and level of social support (F = 92.6, p < 0.001). As a newly developed hypothesis stemming from the scaling analysis, the TRIM-Diabetes Compliance domain was shown to discriminate between the type of treatment (oral vs. other, F = 14.3, p < 0.001). For the TRIM-Device domains, both the Device Function (F = 34.8, p < 0.001) and Device Bother (F = 59.8, p < 0.001) domains distinguished between the fear of injection (D-FISQ).

The relationship between key patient and diabetes characteristics and TRIM-Diabetes and TRIM-Diabetes Device scores can be seen in Table 5. As expected, HbA1c levels had the most consistently significant relationship to TRIM impacts. Additionally, treatment type had a significant relationship to the Total as well as all domains on the TRIM-Diabetes and age and type of diabetes were significant in specific domains in both measures.

**Table 5:** TRIM-Diabetes and TRIM-Diabetes Device Scores by Key Patient Characteristics

**TRIM-Diabetes**						
	**TRIM Burden**	**TRIM Daily Life**	**TRIM Diabetes Management**	**TRIM Compliance**	**TRIM Psychological**	**TRIM Total Score**

**Gender**						

Female (n = 267)	67.5 (22.4)	69.6 (23.5)	58.1 (24.2)	72.6 (22.4)	67.1 (24.0)	66.9 (18.1)

Male (n = 240)	67.7 (21.8)	66.5 (24.4)	56.6 (23.2)	74.4 (23.7)	66.8 (23.4)	66.2 (16.9)

Sig. (p-value)	0.946	0.142	0.471	0.396	0.859	0.672

**Age**						

18-30 (n = 96)	62.7 (24.2)	58.4 (27.8)	60.4 (23.5)	61.7 (26.8)	59.1 (25.3)	60.3 (17.7)

31-60 (n = 227)	67.0 (22.5)	68.3 (23.5)	56.7 (25.1)	72.7 (22.5)	65.2 (24.3)	65.7 (18.0)

61-80 (n = 184)	70.9 (20.1)	73.1 (20.6)	56.7 (22.0)	80.6 (18.5)	73.2 (20.3)	70.8 (15.7)

Sig. (p-value)	0.012	0.000	0.399	0.000	0.000	0.000

**HbA1c**						

< 6.8 (n = 107)	74.0 (20.2)	76.3 (21.5)	65.8 (21.6)	80.3 (18.1)	76.7 (21.1)	74.6 (16.4)

6.8 - 8.0 (n = 117)	66.7 (21.4)	68.3 (21.5)	52.8 (20.8)	76.8 (19.5)	67.2 (21.1)	66.1 (14.5)

> 8.0 (n = 101)	59.1 (22.9)	56.2 (24.8)	49.4 (23.4)	61.9 (25.2)	54.1 (22.7)	55.8 (15.5)

Sig. (p-value)	0.000	0.000	0.000	0.000	0.000	0.000

**Diabetes Type**						

Type 1 (n = 134)	67.4 (23.3)	63.8 (25.6)	60.3 (23.4)	69.4 (25.5)	64.8 (25.2)	65.0 (17.6)

Type 2 (n = 373)	67.7 (21.7)	69.7 (23.1)	56.4 (23.8)	74.9 (21.9)	67.7 (23.1)	67.1 (17.5)

Sig. (p-value)	0.923	0.014	0.096	0.016	0.219	0.250

**Treatment Type**						

Syringe/vial (n = 100)	58.9 (24.1)	64.0 (23.8)	50.1 (25.3)	71.2 (24.6)	63.3 (23.9)	61.3 (18.9)

Insulin Pen (n = 102)	61.4 (22.2)	65.7 (21.8)	50.2 (21.1)	72.1 (22.3)	61.1 (21.5)	61.6 (16.1)

GLP-1 (n = 102)	66.7 (20.8)	63.1 (24.9)	60.8 (24.8)	66.6 (25.8)	63.9 (25.7)	64.2 (16.8)

Insulin pump (n = 101)	71.3 (19.7)	65.4 (23.7)	60.7 (20.5)	73.6 (21.7)	67.0 (22.3)	67.5 (15.8)

Oral/tablet (n = 102)	79.6 (17.2)	82.5 (19.9)	65.1 (23.0)	83.7 (16.7)	79.5 (20.6)	78.1 (14.3)

Sig. (p-value)	0.000	0.000	0.000	0.000	0.000	0.000

**Treatment Type**						

All Insulins(n = 303)	63.9 (22.6)	65.0 (23.0)	53.6 (22.9)	72.3 (22.8)	63.8 (22.6)	63.4 (17.2)

GLP-1 (n = 102)	66.7 (20.8)	63.1 (24.9)	60.8 (24.8)	66.6 (25.8)	63.9 (25.7)	64.2 (16.8)

Sig. (p-value)	0.273	0.467	0.008	0.035	0.973	0.705

**TRIM-Diabetes Device**						

	**TRIM Device Function**		**TRIM Device Bother**		**TRIM Device Total Score**	

**Gender**						

Female (n = 231)	76.3 (19.1)		77.8 (25.1)		76.9 (17.8)	

Male (n = 181)	74.4 (18.7)		76.0 (25.4)		75.0 (16.4)	

Sig. (p-value)	0.305		0.472		0.271	

**Age**						

18-30 (n = 95)	71.5 (19.7)		63.0 (27.3)		68.3 (18.1)	

31-60 (n = 185)	75.7 (19.0)		78.2 (25.4)		76.7 (17.2)	

61-80 (n = 132)	78.0 (17.9)		85.4 (18.2)		80.8 (14.4)	

Sig. (p-value)	0.036		0.000		0.000	

**HbA1c**						

< 6.8 (n = 80)	77.7 (16.6)		78.5 (23.4)		78.0 (15.4)	

6.8 - 8.0 (n = 102)	76.9 (18.7)		81.1 (24.0)		78.5 (17.6)	

> 8.0 (n = 94)	70.0 (21.6)		67.6 (27.8)		69.1 (18.2)	

Sig. (p-value)	0.013		0.001		0.000	

**Diabetes Type**						

Type 1 (n = 133)	75.6 (18.8)		71.1 (27.1)		73.9 (17.6)	

Type 2 (n = 279)	75.4 (19.0)		79.9 (23.7)		77.1 (16.9)	

Sig. (p-value)	0.902		0.001		0.082	

**Treatment type**						

Syringe/vial (n = 100)	76.2 (20.3)		79.6 (25.2)		77.5 (16.9)	

Insulin Pen (n = 102)	74.2 (18.1)		77.3 (22.5)		75.4 (17.4)	

GLP-1 (n = 102)	75.0 (20.6)		76.0 (27.2)		75.4 (17.3)	

Insulin pump (n = 101)	76.2 (16.9)		74.2 (26.0)		75.5 (17.5)	

Sig. (p-value)	0.937		0.656		0.897	

**Treatment type**						

All Insulin (n = 303)	75.5 (18.5)		77.0 (24.6)		76.1 (17.3)	

GLP-1 (n = 102)	75.0 (20.6)		76.0 (27.2)		75.4 (17.3)	

Sig. (p-value)	0.969		0.939		0.935	

#### Interpretability: Minimally Important Difference

For the Burden domain, the mean difference of scores between "Slightly burdensome" and "Somewhat burdensome" on the overall item is 10.6 points. The standard deviation of the higher (lower impact) score is 19.0. One-half of this standard deviation is 9.5 points. As specified in the SAP, using the 1/2 SD criteria, the Burden score difference meets this MID threshold. This same pattern is seen with each of the TRIM-Diabetes domains: Daily Life (Δ = 16.0, 1/2 SD = 9.2); Diabetes Management (Δ = 12.0, 1/2 SD = 8.2); Psychological (Δ = 17.8, 1/2 SD = 8.7); and finally the TRIM-Diabetes Total score (Δ = 17.6, 1/2 SD = 7.8). As Compliance was a new domain, there was no overall item to examine the MID for this domain. For the TRIM-Device Function and Bother domains, the differences did not meet the 1/2 SD thresholds.

#### Final Measures

**Figure 1:**
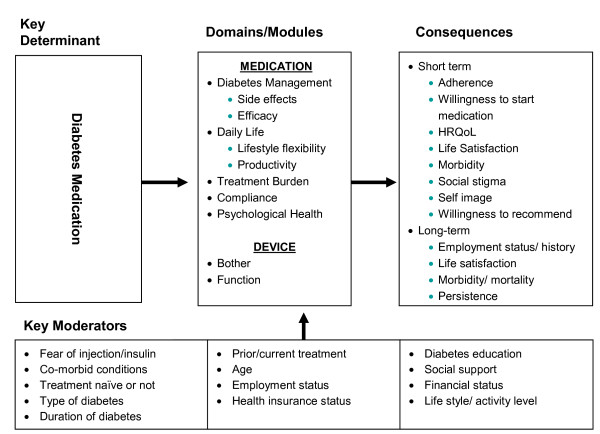
**Conceptual Model**.

Based on the preliminary conceptual model used for item generation and results from the psychometric analyses, a final conceptual model of the impact of diabetes treatment was developed. The Conceptual Model is included as Figure 1.

**Figure 2:**
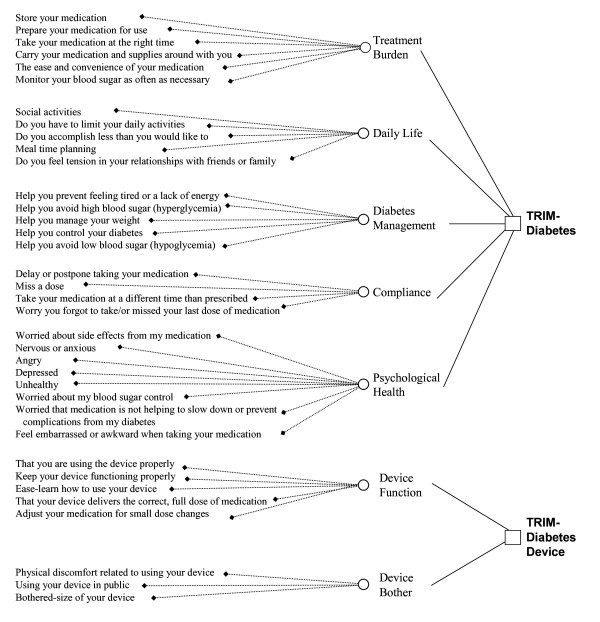
**Conceptual Framework**.

Based on the findings from both Phase 1 and Phase 2 of the study, a 28-item TRIM-Diabetes and an eight-item TRIM-Diabetes Device were finalized. The conceptual framework of items per conceptual domain for each measure is shown in Figure 2.

#### Response Burden

Response burden was imputed from the respondents' recorded time to complete the TRIM-Diabetes/Device. The time for completion of the 28-item TRIM-Diabetes is approximately five minutes and approximately one minute for the TRIM-Diabetes Device or approximately six minutes for the combined TRIM- Diabetes/Device.

## Discussion

The TRIM-Diabetes and TRIM-Diabetes Device measures were developed to create new PRO measures assessing the key impacts of diabetes medication and to be applicable to all treatment delivery modes currently available. These measures are intended to capture the full spectrum of impacts of treatment and cannot be classified as strictly HRQoL or as treatment behavior and satisfaction measures. We believe these concepts interact and influence each other to such an extent that they should be combined to fully understand the broad-spectrum impact of treatment drug and delivery method. Thus, the TRIM-Diabetes/Device are valid and reliable PRO measures to assess the total impact, as well as the specific important domains of impact, some of which more closely represent an HRQoL domain (e.g., Daily Life), while others treatment satisfaction domains (e.g., Treatment Burden), or treatment behavior (Compliance). We suggest that the total score as well as domain subscale scores be referred to as measures of treatment impact. The concept of "impact" may be a more relevant and useful umbrella term for real world treatment decisions and for distinguishing between treatment outcomes.

Certain limitations of the study should be noted. First, the validation study was web-based and may have a respondent selection bias based on access to the internet. We do not believe this bias to be significant, even given the age of participants, as it has been shown that computer use for those over the age of 50 in U.S. is increasing and approximately 75% of people in this age group have computers at home [16]. Further, the comparability of online testing to paper and pencil forms has been shown to be equivalent in psychometric properties [17-19]. Second, we did not have longitudinal data to examine change found with the TRIM-Diabetes; self-report items found elsewhere in the validation battery were used as anchors to approximate the minimally important difference. This analysis is exploratory and meant to provide preliminary estimates of differences established using an anchor-based approach. Since longitudinal data is not being used, one must be cautious in the interpretation of the results in relation to minimally important differences. Additionally, as expected, there was an impact in all scores for both type 1 and type 2 patients and the magnitude of these impacts were not significantly different between diabetes types for the total scores or most domains. The exceptions to this were the TRIM-Diabetes Daily Life and Compliance domains and the TRIM-Device Bother domain, with type 1 patients having a significantly more negative impact. Future research would be helpful to better understand these differences. Differences in the magnitude, rather than type of impacts, may also exist between insulin and the newer GLP-1 analogues, especially in relationship to rates of hypoglycaemia. This is supported by our findings that GLP-1 analogue group, when compared to insulin users, had significantly more positive impacts for Diabetes Management, the domain which captures the impact of hypoglycaemia, as well as in the Compliance domain. This suggests that the absence of hypoglycaemic episodes may increase compliance with treatment, a finding which should also be explored in future research. The absence of significant differences between treatment types and scores on the TRIM-Diabetes Device are concerning and require further examination. Finally, the use of the TRIM-Diabetes in non-English speaking countries or in subgroups of patients known to have characteristics which may influence PROs such as the impact of diabetes complications on treatment satisfaction should be examined in future studies. Validation is an iterative process and this study represents the first step in that process. Future validation work is planned for the TRIM-Diabetes/Device measures which will confirm the factor structure, examine responsiveness in a clinical trial population and explore the relationship of the measures to other clinical factors.

## Conclusion

The TRIM-Diabetes and the TRIM-Diabetes Device have been found to have acceptable psychometric properties and can be considered well-developed and validated PRO measures. Treatment and device-specific measures, such as these TRIM measures, should have greater face validity, be more responsive to change over time, and may be more useful to both clinicians and researchers to assess the impact of diabetes treatment. This comprehensive understanding of impacts should provide valuable insight to health care professionals and facilitate physician-patient interactions, improve adherence and persistence of treatments, and lead to management plans tailored to the individual.

## Competing interests

MB, SL and DB are paid consultants to the pharmaceutical industry. TC and MH are employees of Novo Nordisk who sponsored this project.

## Authors' contributions

MB designed the study, conducted all interviews, assisted in the analysis and drafted the manuscript. MH and TC assisted in the design of the study and in the drafting of the manuscript. SL assisted in the conducting of interviews, analysis of the data and the drafting of the manuscript. DMB prepared the analysis plan, conducted the analyses and assisted in the preparation of the manuscript. All authors read and approved the final manuscript.
